# First Complete Genome Sequence of a Subdivision 6 *Acidobacterium* Strain

**DOI:** 10.1128/genomeA.00469-16

**Published:** 2016-05-26

**Authors:** Sixing Huang, Selma Vieira, Boyke Bunk, Thomas Riedel, Cathrin Spröer, Jörg Overmann

**Affiliations:** Leibniz Institute DSMZ-German Collection of Microorganisms and Cell Cultures, Braunschweig, Germany

## Abstract

Although ubiquitous and abundant in soils, acidobacteria have mostly escaped isolation and remain poorly investigated. Only a few cultured representatives and just eight genomes of subdivisions 1, 3, and 4 are available to date. Here, we determined the complete genome sequence of strain HEG_-6_39, the first genome of *Acidobacterium* subdivision 6.

## GENOME ANNOUNCEMENT

Bacteria belonging to the phylum *Acidobacteria* represent one of the dominant groups of soil bacteria, constituting an average fraction of 20% in 16S rRNA gene libraries (1). Despite their ubiquitous distribution and abundance, only a few *Acidobacteria* have so far been isolated in pure culture. As a result, little is known about their physiology and ecological functions in soil. Currently, 26 subdivisions have been proposed (2), and among them, subdivisions 1, 3, 4, and 6 are the most abundant in soils worldwide (1, 3). Prior to this study, no successful isolation of subdivision 6 acidobacteria had been reported.

Strain HEG_-6_39 was isolated from grassland soil in the German Biodiversity Exploratories (4). Genome sequencing was carried out on the PacBio RSII (Pacific Biosciences, Menlo Park, CA) using P6 chemistry. Genome assembly was performed with the “RS_HGAP_Assembly.3” protocol included in the SMRT Portal version 2.3.0, utilizing 97,934 postfiltered reads, with an average read length of 13,738 bp. One complete chromosomal contig was obtained and trimmed, circularized, and adjusted to *dnaA* (*locustag*_00001) as the first gene. A final genome quality of QV60 was determined during resequencing using the RS_BridgeMapper.1 protocol in SMRT Portal. In addition, genome sequencing was carried out on a HiSeq 2500 (Illumina, San Francisco, CA) in a 100-bp paired-end single-indexed run, resulting in 3.2 million paired-end reads. Quality improvement was performed with the Burrows-Wheeler Aligner (BWA) (5), mapping the Illumina reads onto the obtained chromosome. Protein-coding regions, 16S rRNA, and tRNA genes were scanned and annotated with Prokka (6). The genome was also uploaded to the RAST (7) service for comparative analysis. Metabolic pathways were identified online at KEGG (8), with subsequent manual gap filling.

The genome contained 7,480,314 bp and 6,295 predicted protein-coding genes. The G+C content was 64.7%. Only one rRNA operon was found. In addition, like other bacterial genomes, one transfer-messenger RNA (tmRNA) gene was found. The RAST annotation recognized one-third of the coding sequences (CDSs) as subsystem related. The most populated subsystem categories were carbohydrates (401), amino acids and derivatives (389), and RNA metabolism (184).

It is noteworthy that the genome contains 104 glycoside hydrolases and 183 peptidases. The complete gene sets for assimilatory nitrate reduction and sulfite reduction were found. Also identified are detoxification operons against arsenate, arsenite, antimonite, cobalt, zinc, lead, cadmium, and mercury. Antimicrobial resistance genes, such as *mdtABC* drug exporter genes, were also found. The HEG_-6_39 genome contains three cold-shock protein genes, *cspADE*, and two pathways for trehalose biosynthesis.

The availability of high-quality genome sequences for *Acidobacteria*, particularly those belonging to subdivision 6, will improve our understanding of the functional implication of these organisms in the soil environment.

### Nucleotide sequence accession number.

The nucleotide sequence has been deposited at GenBank under the accession no. CP015136.
